# Effects of different applied voltages of irreversible electroporation on prostate cancer in a mouse model

**DOI:** 10.1038/s41598-022-25258-3

**Published:** 2022-12-26

**Authors:** Hong Bae Kim, Chu Hui Zeng, Yunlim Kim, Seung Jeong, Song Hee Kim, Jeon Min Kang, Yubeen Park, Dong-Sung Won, Ji Won Kim, Dae Sung Ryu, Bumjin Lim, Jung-Hoon Park

**Affiliations:** 1 Department of Biosystems & Biomaterials Science and Engineering, Seoul National University, Seoul, 08826 Republic of Korea; 2 Biomedical Engineering Research Center, Asan Medical Center, Asan Institute for Life Sciences, 88 Olympic-Ro 43-Gil, Songpa-Gu, Seoul, 05505 Republic of Korea; 3 Department of Urology, Asan Medical Center, University of Ulsan College of Medicine, 88 Olympic-Ro 43-Gil, Songpa-Gu, Seoul, 05505 Republic of Korea

**Keywords:** Diseases, Medical research, Oncology

## Abstract

As a non-thermal ablation method, irreversible electroporation (IRE) has been widely investigated in the treatment of prostate cancer. However, no consensus has been achieved on the optimal parameters of IRE for prostate cancer. Since high voltage is known to carry risks of muscle contraction and patient discomfort, it is crucial to identify the minimum but effective and safer applied voltage to inhibit tumor growth. In this study, the effect of different applied voltages of IRE on prostate cancer was evaluated in BALB/c nude mice. Mathematical simulation and measurement of the actual ablation area revealed a larger ablation area at a higher voltage. In in vivo experiment, except for the three different voltages applied, all groups received identical electrical conditions: pulse number, 180 (20 groups × 9 pulses/group); pulse width, 100 µs; pulse interval, 2 ms; distance between the electrodes, 5 mm; and electrode exposure length, 15 mm. Whilst the tumor volume initially decreased in the 500 V (1000 V/cm) and 700 V (1400 V/cm) groups and subsequently increased, only a transient increase followed by a continuous decrease until the sacrifice was observed in the 900 V (1800 V/cm) group. This result demonstrated a lasting effect of a higher applied voltage on tumor growth inhibition. The histological, immunohistochemical, and western blot findings all confirmed IRE-induced apoptosis in the treatment groups. Taken together, 900 V seemed to be the minimum applied voltage required to reduce tumor growth, though subsequent studies are anticipated to further narrow the voltage intervals and lower the minimum voltage required for tumor inhibition.

## Introduction

As the second leading cause of cancer-related deaths in men, prostate cancer threatens patients’ lives and poses a severe healthcare burden worldwide^1^. Current treatment options include radical prostatectomy, radiotherapy, and various minimally invasive ablation options^2^. Unfortunately, radical prostatectomy, the conventional surgical technique that aims to resect the cancerous tissues to improve the survival rate, can be too aggressive, cause serious side effects such as urinary incontinence and sexual dysfunction, and impair patients’ quality of life^3^. In contrast, even with the potential to minimize patient discomfort, minimally invasive local treatments, including radiofrequency and microwave ablation, cryoablation, high-intensity focused ultrasound, vascular-targeted photodynamic therapy, interstitial laser thermotherapy, brachytherapy, and stereotactic radiotherapy, remain investigational at best, with rather insufficient long-term prospective comparative data being available^2^.

In recent years, irreversible electroporation (IRE) has attracted tremendous attention for the treatment of various cancers, owing to its unique characteristics^4–6^. Unlike microwave or radiofrequency ablation, IRE is thermal-independent that does not involve the use of heat to destroy cancer cells. Instead, it creates nano-scale pores in the cell membrane, increasing permeability, causing loss of homeostasis, and eventually leading to cell death^7,8^. Another advantage of IRE is its ability to protect the extracellular matrix and not be affected by the heat sink effect, a demerit of thermal ablation. In addition, considering the anatomical distance between the prostate and heart, cardiac arrhythmias can hardly occur when using IRE for prostate cancer. Therefore, IRE is becoming increasingly popular in the treatment of prostate cancers as well as other tumors. The performance of IRE highly depends on the combination of four pulse parameters, namely, pulse repetition, length, interval, and magnitude of applied voltage^9^. However, despite the increasing number of investigations on IRE, achieving consensus on the optimal IRE parameters is challenging, and the parameters vary largely across the studies. In particular, for a given set of pulse parameters, the electric field, which is controlled by the applied voltage and electrode spacing, is the primary factor in defining the spatial distribution of cell death. Moreover, finding the appropriate electric field strength (i.e., voltage-to-distance ratio) for IRE means critical as a sufficiently strong electric field strength ensures the destruction of the targeted lesion while preserving as much of the healthy tissues as possible^10^. Therefore, the purpose of the study was to compare the efficacy of different applied voltages, while keeping the electrode spacing consistent across the animals, for treating prostate cancer in a nude mouse model.

## Materials and methods

### Cell culture

The human prostate cancer cell line (DU145) was purchased from Korean Cell Line Bank (Pohang, Korea). The cells were cultured in Roswell Park Memorial Institute 1640 medium with L-glutamine (300 mg/L), 25 mM HEPES, and 25 mM NaHCO_3_ and supplemented with 10% fetal bovine serum (FBS; Sigma-Aldrich, St. Louis, MI, USA), 100 μg/mL streptomycin, and 100 U/mL penicillin at 37 ℃ in 5% CO_2_ atmosphere. Before each implantation procedure, the viability of the cells was tested through trypan blue staining (confirming > 90% cell viability for each tumor implantation procedure).

### Animal study design

**Figure 1:**
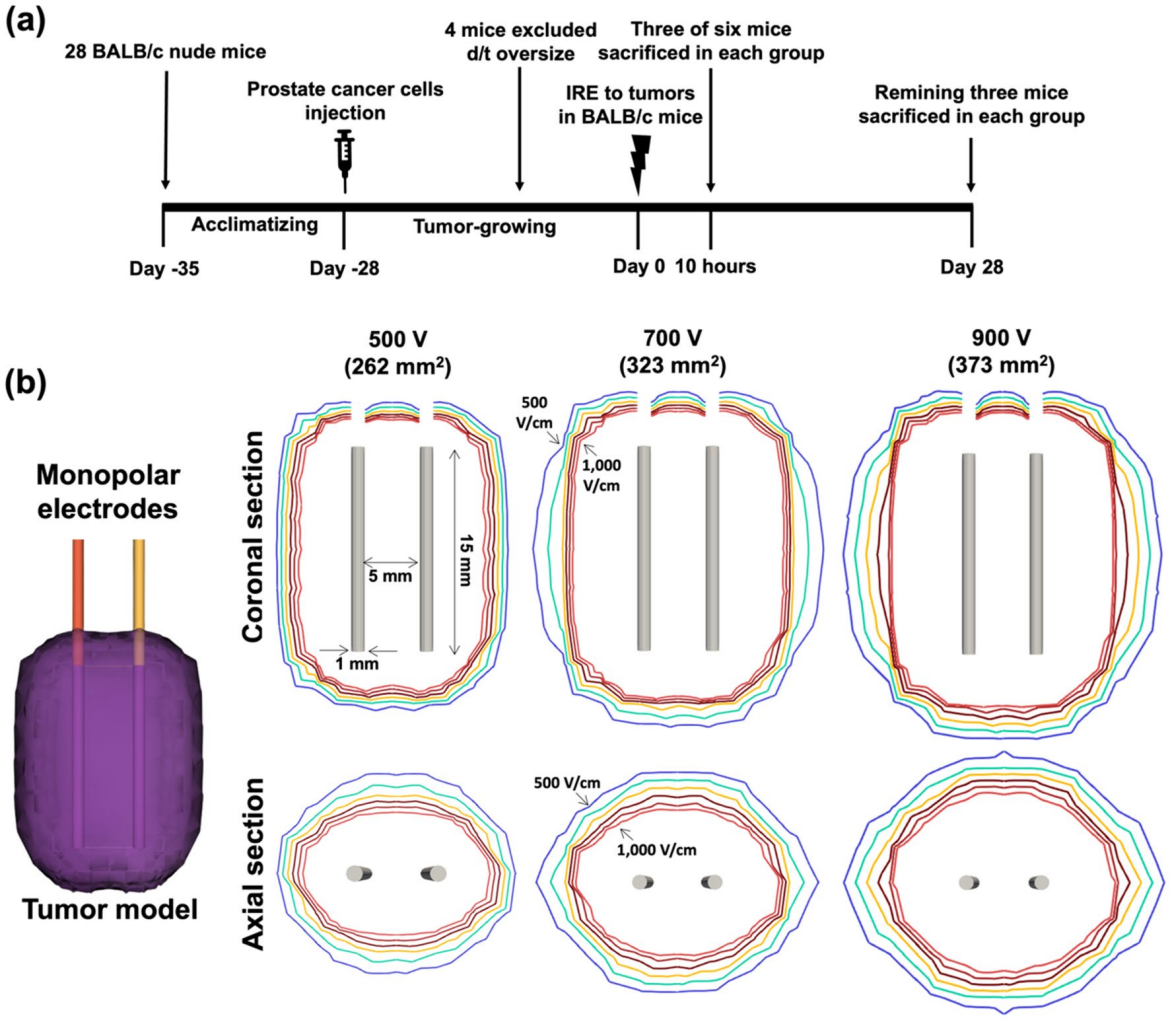
In vivo schedule and study design and simulation of electrical field strength. (**a**) In vivo study scheme with irreversible electroporation (IRE) procedure. (**b**) Mathematical simulation showing an increasingly larger ablation area with a climbing applied voltage of IRE. While the highest field strength (1000 V/cm) generated a relatively consistent ablation area at all applied voltages, the potential at a weaker field strength tended to expand outwards at higher voltages, producing a larger ablation area overall.

This study was approved by the Institutional Animal Care and Use Committee of Asan Medical Center (#2020-12-238) and conformed to the US National Institutes of Health guidelines for handling laboratory animals. The study was carried out in compliance with the ARRIVE guidelines. The overall study scheme is presented in Fig. 1. All animals were housed under a 12 h light/dark cycle at environmental temperature (24 ± 1 ℃) and humidity (55 ± 10%). Three mice were housed per cage with free access to food and water. Twenty-eight 5-week-old male BALB/c nude mice (Central Lab. Animal Inc., Seoul, Korea) weighing 15–20 g were acclimatized for 7 days before injecting the human prostate cancer cell lines. Prostate cancer cells (passage 15) of 3.0 × 10^6^ of 0.1 mL containing Matrigel were injected subcutaneously into the right flank of each mouse and allowed to grow for 4 weeks^11^. Four weeks after cancer cell implantation, the tumor volume was calculated using the formula:

1 $$ {\text{V }} = \, \left( {{\text{W}}^{{2}} \times {\text{ L}}} \right)/{2} $$

for caliper measurements, where W is the tumor width and L is the tumor length^12^. The tumor enlarged above 1000 mm^3^ in 4 of the 28 (14.3%) mice, and these four mice were excluded from the subsequent IRE procedure owing to oversized tumors. Finally, 24 (85.7%) mice were enrolled in this study and randomly distributed into four groups with six in each: the control group, which received no applied voltage but electrode penetration only; the 500 V group, in which a voltage of 500 V was applied; the 700 V group; and the 900 V group. Randomization was conducted by a researcher who was blinded to the detailed data of the 24 mice. For each group, three of the six mice were sacrificed 10 h after the IRE procedure, and the remaining three mice were sacrificed at 4 weeks by inhalation of pure carbon dioxide.

### Irreversible electroporation devices

To deliver electrical energy, 19 G monopolar electrodes (The Standard Co., Ltd., Gunpo-si, Korea) made of stainless steel were introduced into the tumor tissue. The needle-type electrodes had a diameter of 1 mm and an exposed length of 15 mm, while the rest was insulated using a thermal tube. The electrodes were inserted 5 mm apart. An electrical generator (EPO-S1, The Standard Co., Ltd.) capable of generating up to 3000 V (current, 50 A; pulse width, 100 µs [range, 100–1000 µs]; and pulse interval range, 100–2000 µs) was used for IRE.

### Simulation of electrical field strength

The applied electric fields were simulated using the open-source OpenFOAM software (The Standard Co., Ltd.) to identify the distribution of the electric field strength in each group. The electric field distribution was obtained from the Laplace equation,

2 $$ \nabla^{ \wedge } 2\Phi = 0 $$

where Φ denotes the electric potential. For the simulation, the prostatic tissue was modeled as a cylinder with a height and diameter of 80 mm × 20 mm, respectively. The exposed solid electric surface was treated with an electric boundary condition: Φ = V (source) and Φ = 0 (sink); the other surfaces were defined to be electrically insulated. Mesh generation for the simulation was completed using a snappy hex mesh instead of a block mesh. The mesh cells were 93,078 in volume. The ablation area was calculated at the equipotential of 500 V/cm using ImageJ (version 1.53f51; National Institutes of Health, USA)^10^.

### Irreversible electroporation procedure

Anesthesia was induced through an intramuscular injection of 50 mg/kg zolazepam and tiletamine (Zoletil 50; Virbac, Carros, France) and 10 mg/kg xylazine (Rompun; Bayer HealthCare, Leverkusen, Germany). IRE was then performed in the mice using the corresponding voltages. The electrodes were aligned along the axis of the largest tumor diameter to ensure maximal ablation of the tumor. For corroboration of the thermal effect during the IRE procedure, the temperature was monitored every 250 ms using a thermometer (FTX-100-LUX + , OSENSA Innovations Co., Burnaby, BC, CA) using a fiber with a transmitter (PRB-G20-2.0 M-ST-C, OSENSA Innovations Co.). To measure the current passing through the electrodes, an oscilloscope (TDA 3044B, Tektronix, USA) was used. To measure the electrical conductivity of tumor tissues before and after the IRE treatment, an impedance analyzer (4192A, Hewlett Packard, USA) was used at a frequency of 10 Hz^13^. Except for the three different voltages applied, all groups received identical and consistent electrical conditions: pulse number, 180; pulse width, 100 µs; pulse interval, 2 ms; distance between the electrodes, 5 mm; and electrode exposure, 15 mm. To decrease the thermal effect, group pulses (pulse width, 100 µs; pulse interval, 2 ms; and group pulse interval, 2 s) were split into 20 groups with 9 pulses per group^9^.

### Tumor volume and weight analysis

The tumor volume and body weight were measured at 1, 3, 7, 12, 14, 21, and 28 days after the procedure. The length and width of the tumor were measured using Vernier calipers (Mitutoyo, Japan), and volumetric analysis was performed using the formula:

3 $$ V = \pi /6 \times f \times \left( {length \cdot width} \right)^{3/2} $$

where the constant *f* was 1.69 for BALB/c mice^14^. On day 28, the tumor was extracted and weighed following the sacrifice of the animals.

### Histological examination

The extracted tumor tissues were fixed in 10% neutral buffered formalin for 24 h and embedded in paraffin. The IRE-ablated tumor tissues were sectioned transversely for microscopic examinations. The slides were stained with hematoxylin and eosin (H&E) for histological analysis. Immunohistochemistry was performed on paraffin-embedded sections using terminal deoxynucleotidyl transferase-mediated dUTP nick and labeling (TUNEL; S7100, ApopTag Peroxi-199 dase, Sigma Aldrich, St. Louis, MO, USA) primary antibodies to verify apoptosis after the IRE procedure and to measure the ablation area. Digitalized slides were viewed using a scanner (Pannoramic 250 FLASH III, 3D HISTECH Ltd., Budapest, Hungary), and the ablation area was analyzed using a viewer software (CaseViewer, 3D HISTECH Ltd).

### Western blotting

For western blotting, the tumors were isolated from the mice. To prepare tissue lysates, tumor tissues were transferred to a RIPA lysis buffer containing 50 mM Tris–HCl (pH 7.8), 150 mM NaCl, 1% IGEPAL, 10 mM NaF, 0.1 mM EDTA, and a protease inhibitor cocktail (Sigma Aldrich). Homogenization was then performed using sonication in the buffer. The prepared tissue lysate was micro-centrifuged at 13,000 × g for 20 min. Then, the protein concentration was measured using the Bradford protein assay (Bio-Medical Science Co., Ltd, Seoul, Korea). The tissue lysate was mixed with the same amount of protein (20 µg in 15 µL) as 5 × sodium dodecyl sulfate (SDS) loading sample buffer. The tissues were heated at 100 ℃ for 7 min before loading and then separated on precast 4–20% gradient SDS polyacrylamide gels (Bio-Medical Science Co., Ltd). After the proteins were transferred to a polyvinylidene fluoride membrane (Merck Millipore, Burlington, MA, US), nonspecific binding to the membrane was blocked for 1 h at room temperature with 5% bovine serum albumin (BSA) in tris-buffered saline with Tween-20, and the membrane was incubated with antibodies against cleaved caspase-3 (1:1000, Cell Signaling Technology, Danvers, MA, US) overnight at 4 ℃ in a shaking inhibitor (110 rpm orbital shaker). β-actin (1:10,000, Santa Cruz Biotechnology, Dallas, TX, US) was used as an internal control. Then, the membrane was incubated with horseradish peroxidase-conjugated IgG secondary antibodies in 5% blocking buffer (nonfat milk or BSA), with shaking for 1 h at room temperature. Protein bands appeared after the enhanced chemiluminescent solution (Merck Millipore) was added. The quantitative intensity of the proteins was normalized to β-actin and depicted in histograms.

### Statistical analysis

Data are presented as mean ± standard deviation (SD). Statistical significance between the groups was determined using either a two-tailed paired Student’s *t*-test (significance, * *P* < 0.05; ** *P* < 0.01; *** *P* < 0.001) or a one-way ANOVA (significance, # *P* < 0.05; ## *P* < 0.01) followed by Tukey’s post hoc analysis. Statistical analyses were performed using SPSS (version 27.0; IBM Corp., Armonk, NY, USA).

## Results

### Mathematical simulation

The mathematical simulation of the IRE is presented in Fig. 1. The simulated ablation area was displayed coronally and axially with equipotential lines outlining the electric field strength of 500–1000 V/cm at an increment of 100 V/cm. As the applied voltage increased, the ablation area became increasingly larger, and the total ablation area measured on the coronal plane was 262 mm^2^, 323 mm^2^, and 373 mm^2^ at 500 V, 700 V, and 900 V, respectively. Moreover, while the highest field strength (1000 V/cm) maintained a relatively consistent ablation area at all applied voltages, the potential at a weaker field strength, especially at 500 and 600 V/cm, tended to expand outwards at higher voltages and produced larger ablation areas.

### Procedural outcomes

**Figure 2:**
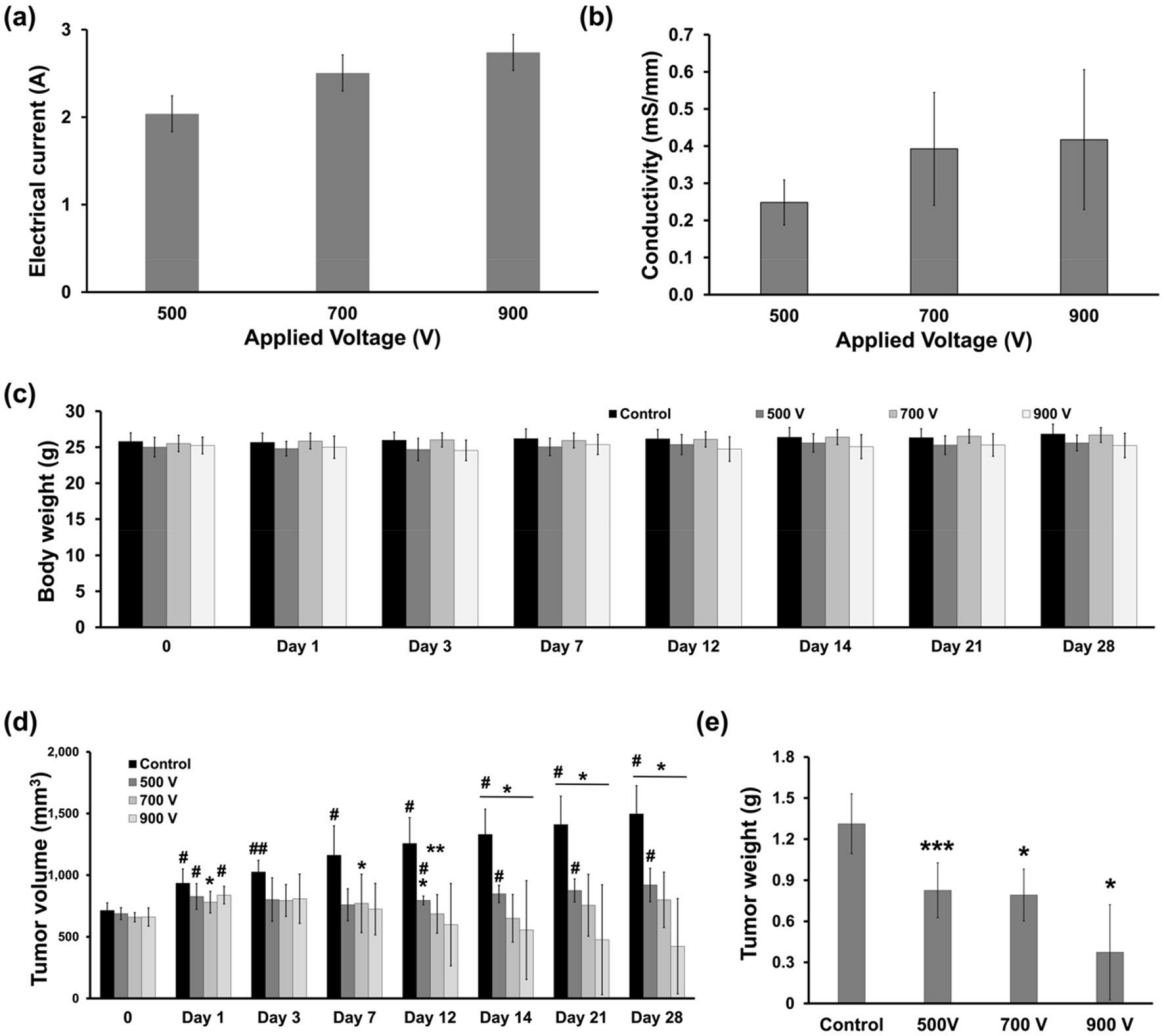
Safety measures of the irreversible electroporation procedure. (**a**) Electrical current and (**b**) change in conductivity increased proportionally with the applied voltages. (**c**) The body weight did not significantly change in any of the groups. (**d**) Except for the control group in which the tumor volume kept increasing, all the treatment groups experienced an increase and then a decrease in the tumor volume. The 900 V group seemed to be the only group witnessing a lasting inhibition of tumor volume. (**e**) The higher voltage generated lower tumor weight at post-procedural day 28.

The procedure was successfully performed in the enrolled 24 mice without procedure-related complications. All animals subjected to IRE recovered spontaneously and survived until the assigned follow-up date. The average peak electrical currents for 180 pulses increased proportionally with the applied voltages (Fig. 2a). The change in the mean (± SD) conductivity of the tumor tissues was 0.25 ± 0.06 mS/mm in the 500 V group, 0.39 ± 0.15 mS/mm in the 700 V group, and 0.42 ± 0.19 mS/mm in the 900 V group (Fig. 2b). The temperature fluctuated but remained under 40 ℃ during the IRE procedure.

### IRE-induced tumor volume changes

The body weight of the enrolled mice was not significantly different among the groups (Fig. 2c). The tumor volume in the control group increased continuously. Compared to the control group, the tumor volume showed a 0.65-fold decrease from post-IRE Day 1 to Day 7 and remained constant from Day 7 to the end of the study in the 500 V group. Similarly, in the 700 V group, a 0.49-fold decrease in tumor volume was observed from Day 7 to Day 14 (*P* < 0.05) followed by a 0.53-fold increase from Day 14 to the end of the study compared to the control group. In contrast, despite a transient increase from Day 0 to Day 1 in the 900 V group in which necrotic scarring was observed, the tumor volume continuously decreased from Day 1 to the end of the study compared to the control group (0.9-fold and 0.28-fold on Day 1 and Day 20, respectively; both *P* < 0.05). Thus, except for the 900 V group, all other groups were associated with a higher tumor volume compared to the initial value, suggesting the efficacy of higher voltage in inhibiting prostate cancer growth (all *P* < 0.05) (Fig. 2d). Meanwhile, the higher the voltage applied, the lighter the tumor was, with the tumor weight being 1.31 ± 0.22 g in the control group and 0.37 ± 0.35 g in the 900 V group on Day 28 after IRE treatment (all *P* < 0.05) (Fig. 2e).

### Histological findings

**Figure 3:**
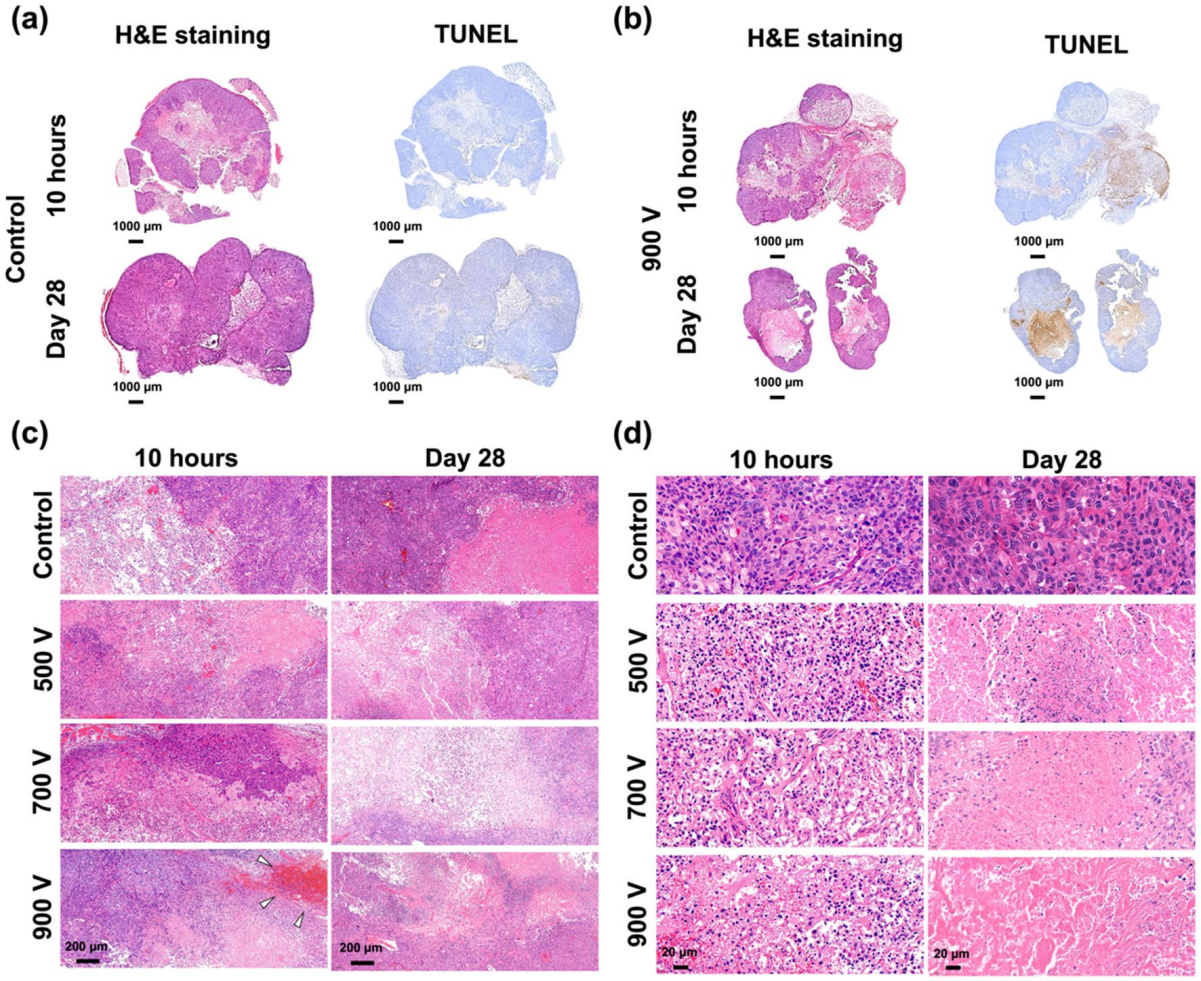
Representative microscopic images of tumor tissue at different applied voltages. (**a**–**b**) Hematoxylin and eosin (H&E) staining and terminal deoxynucleotidyl transferase-mediated dUTP nick and labeling (TUNEL) assay revealed (**a**) natural necrosis at 10 h and 28 days in the control group and (**b**) destructive findings after irreversible electroporation (IRE) ablation in the 900 V group. (**c**) Histological images showing extensive tumor apoptosis and necrosis in all groups and bleeding (arrowheads) in the 900 V group. (**d**) Magnified histological images showing remnant cells in apoptosis and condensed cellular nuclei at post-procedural 10 h and shrunken nuclei but rich fiber tissues at 28 days. Note: H&E, hematoxylin and eosin; TUNEL, terminal deoxynucleotidyl transferase-mediated dUTP nick and labeling.

**Figure 4:**
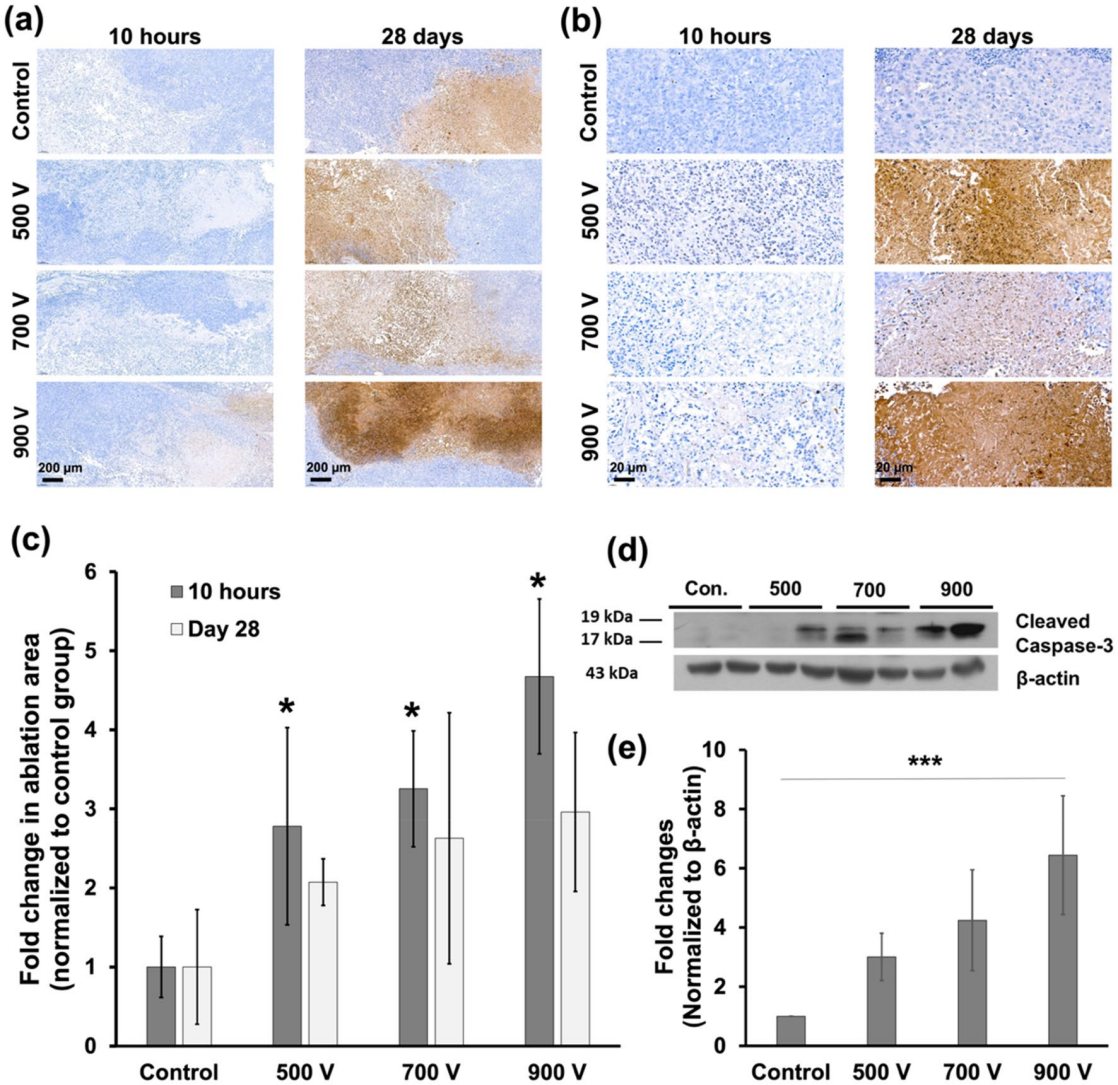
Representative terminal deoxynucleotidyl transferase-mediated dUTP nick and labeling (TUNEL)-stained images and western blot findings at different applied voltages. (**a**–**b**) TUNEL-stained images showing the boundary between non-IRE-treated and IRE-treated regions at (**a**) 10 h and 28 days and (**b**) magnified views. (**c**) The ablation area became proportionally larger as the applied voltage increased. Within the same group, the ablation area at 28 days was smaller than that at 10 h. (**d**) The cleaved caspase-3 band was not detected in the control group and could be seen in the other three groups with an increasingly prominent tendency as the applied voltage increased. (**e**) The expression of cleaved caspase-3 bands showed an approximately 3-, 4.2-, and 6.4-fold increase in the 500, 700, and 900 V groups, respectively, compared to the control group.

Histologic findings are shown in Fig. 3 and 4. The coronally sectioned H&E-stained tissues showed irregular borders in all samples including the control group. Natural necrosis was observed in the control group (Fig. 3a), and samples receiving IRE were found to be destructive at both 10 h and 28 days after IRE (Fig. 3b). Hemorrhage observed immediately after the procedure in the 900 V group was no longer detectable on Day 28 (Fig. 3c). The magnified images in Fig. 3d showed shrinkage of nuclei, empty cavities between nuclei, and unbuilt extracellular matrix compared with the control group. However, necrotized tissues with fewer nuclei were observed at 28 days compared to 10 h after IRE. Also, a few thread-like fibroblasts were seen in the ablated tissues after IRE. As shown in Fig. 4, the TUNEL assay revealed apoptotic tissues on Day 28 after IRE, while only a small part was stained in the 900 V group at 10 h, suggesting a minimum of 10 h required for apoptosis to occur after IRE treatment. One thing to be noted was that although apoptosis was seen in the control group on Day 28, it was likely due to naturally developed apoptosis in the tumor. Then, the apoptotic area was quantitatively assessed based on the TUNEL assay (Fig. 4). The ablation area showed an approximately 2.8-, 3.3-, and 4.7-fold increase in the 500, 700, and 900 V groups, respectively, compared to the naturally developed necrotic area in the control group at 10 h after IRE (all *P* < 0.05) and approximately 2.1-, 2.7-, and 3.0-fold increase at 28 days. The gradual decrease in the ablation areas from post-procedural 10 h to 28 days indicated rare cell recovery after IRE treatment. Here, the difference in the ablation area between the simulated and the mouse model was due to the cut area away from the largest section area.

### Western blot findings

The level of cleaved caspase-3 after IRE was analyzed using western blot to test the extent of apoptosis induced by IRE. As shown in Fig. 4, the cleaved caspase-3 band was not detected in the control group but could be seen in the IRE-treated groups with an increasingly prominent tendency as the applied voltage increased. The cleaved caspase-3 bands were normalized to quantify against β-actin expression, and the expression of cleaved caspase-3 bands showed an approximately 3-, 4.2-, and 6.4-fold increase in the 500, 700, and 900 V groups, respectively, compared to the control group (all *P* < 0.001).

## Discussion

Given IRE’s unique advantages, its use in the treatment of cancers has been widely evaluated. In this present study, the effect of different applied voltages of IRE on prostate cancer was investigated in BALB/c nude mice while keeping the electrode spacing consistent throughout the experiment. Mathematical simulation generated a larger ablation area at a higher voltage, which was later confirmed by measuring the actual ablation area. Electrical current and change in conductivity increased at higher voltages, and temperature remained under 40℃ throughout the experiment. In contrast to the control group in which the tumor volume kept increasing, the tumor volume initially decreased in the 500 V and 700 V groups and subsequently increased until the end of the study; however, it underwent a transient increase followed by a continuous decrease until sacrifice in the 900 V group. The histological, immunohistochemical, and western blot findings all revealed IRE-induced apoptosis in the treatment groups, with only necrosis but no apoptosis being discovered in the control group. Overall, the applied voltage of 900 V demonstrated a lasting effect on the reduction of tumor growth, implying its efficacy for treating prostate cancer without causing serious procedure-related complications.

Various minimally invasive procedures, such as radiofrequency ablation and high-intensity focused ultrasound, have been used for prostate cancer both in animals and humans^15–17^. However, the heat generated by these treatment methods can damage the surrounding healthy tissue, making tumor ablation near the heat-sensitive structures challenging^8^. Compared with these treatment options, IRE possesses several advantages. First, IRE does not usually generate considerable heat during the procedure when its parameters are well controlled. Although Joule heating has been a concern in recent reports, it can be effectively prevented as this heating typically occurs in the immediate vicinity of the electrodes and when too much energy is applied too quickly^18^. A previous study also reported that the temperature rise around the electrodes did not exceed the threshold if the electric pulses were applied for less than 100 μs^9^. The finding in the current study was consistent with the statement that when a pulse width of 100 μs was applied, the temperature measured remained under 40℃ throughout the experiment. From this point, IRE is much safer than other heat-generating procedures, such as high-intensity focused ultrasound, in which heat can bring up the temperature in the exposed tissue to above 60℃ in just 1 s^19^. Second, by creating permanent defects in the cell membrane, IRE is well known for preserving the extracellular matrix of the treated area and sparing the structural integrity of vasculature^20^. This is particularly important for hypervascular structures or lesions such as prostate cancer, for the risk of bleeding can be minimized. Third, though IRE carries a risk of cardiac arrhythmias due to muscle contraction induced by electrical stimulation of excitable cells in the body, this risk is negligible in prostate cancer considering the anatomical distance between the prostate and heart^21^.

Although a few clinical trials have been conducted for prostate cancer, there is yet no consensus on the optimal IRE parameters^22–24^. Previous studies on IRE ablation for prostate used an electric field strength ranging from hundreds to thousands of V/cm. Srimathveeravalli et al*.* found that at the electric field strength of 700 V/cm, the simulation estimated electric field distribution was not different from the ablation defect seen on follow-up magnetic resonance imaging (*P* = 0.43)^25^. In Neal et al*.*’s study, an average of 1135 V/cm electric field strength was created in two patients, and hematuria was identified in both^26^. In contrast, bleeding was only seen in the 900 V group, which was equivalent to 1800 V/cm, in the current study. Another trial investigated by Murray et al*.* used a much higher electric field of 2340 V/cm on average in 27 patients. However, adverse events developed in 14 of them (51.9%) at 6-month follow-up, and positive cancer cells were detected on biopsy^27^. The retrospective analysis on 429 patients undergoing 471 IRE treatments demonstrated the safety, efficacy, and suitability for treating prostate cancer at all clinical stages and recurrent disease, and the use of 1518.13 ± 204.05 V/cm on average was comparable to the finding in the current study. The inconsistent results generated made the proper application of IRE in the clinical setting challenging. However, since electroporation allows ion transportation over the cellular membrane, a considerably high voltage can elicit generalized muscle convulsions and contractions during the treatment, and this explains why general anesthesia is required to ease patient discomfort despite the procedure itself being minimally invasive^20,28^. In addition, increased voltages carry a higher risk of unwanted thermal damage to healthy tissues^29^. Therefore, it is necessary to continue to explore lower but effective applied voltages for the IRE procedure. In the treatment groups of this study, three different voltages, 500 V, 700 V, and 900 V, were compared. With a consistent electrode distance of 5 mm, the electric field strength was therefore 1000 V/cm, 1400 V/cm, and 1800 V/cm, respectively, which were lower than the values reported in previous clinical studies^9^. Although tumor weight was significantly lowered in all three groups compared with that in the control group (all *P* < 0.05), tumor volume continued to decrease only in the 900 V group. Based on these results, it can be concluded that an applied voltage of 900 V, or equivalently, an electric field strength of 1800 V/cm, was the minimum effective applied voltage for prostate cancer in this study. Notably, 1800 V/cm may be a more accurate description for IRE parameters because electric field strength is not only affected by the applied voltage but also by the distance between the electrodes^9^. From this perspective, the electrodes with adjustable distances might represent a future direction for the advancement of IRE.

A few limitations to the study need to be addressed. First, the tumor volume might have been too large for the mice. This was evidenced by the much larger necrotic area in the control group than in the treatment groups. Second, given the limited number of animals in each group, it was difficult to recruit more detailed time points for histological examinations between the period immediately after the procedure and 28 days. Third, the interval between the tested voltages was quite large (i.e., 200 V). This study demonstrated that 900 V was the most effective voltage, compared to the other lower voltages, but it was somehow uncertain whether 900 V was the actual minimum applicable voltage that could produce a large necrotic area in the tumor. Also, since more bleeding was observed in the 900 V group, exploring lower and safer applied voltages is worth further investigation, and the effects of varying other parameters (e.g., pulse length, pulse number, etc.), electrode array, and the number of ablation sessions on treatment outcomes also deserve much attention.

In summary, this study successfully demonstrated the effect of different applied voltages on prostate cancer in a BALB/c nude mouse model. Compared with the other two voltages, 900 V was the minimum voltage required to produce a lasting effect on inhibiting tumor growth. Considering the relatively wide interval between the applied voltages, more detailed studies investigating the minimum required applied voltage are warranted.

## Supplementary Information

Supplementary Information.

## Data Availability

The authors confirm that the data supporting the findings of this study are available within the article. Raw data that support the findings of this study are available from the corresponding authors upon reasonable request.
