# Supplementary material for: Effects of different applied voltages of irreversible electroporation on prostate cancer in a mouse model
Source: Sci Rep. 2022 Dec 26;12:22336. doi: 10.1038/s41598-022-25258-3 (PMC9792528; doi:10.1038/s41598-022-25258-3)
Supplement: Supplementary file 1 — Supplementary Information. [file 41598_2022_25258_MOESM1_ESM.docx]

**Effects of Different Applied Voltages of Irreversible Electroporation on Prostate Cancer in a Mouse Model**

Hong Bae Kim^1†^, Chu Hui Zeng^2†^, Yunlim Kim^3^, Seung Jeong^1^, Song Hee Kim^2^, Jeon Min Kang^2^, Yubeen Park^2^, Dong-Sung Won^2^, Ji Won Kim^2^, Dae Sung Ryu^2^, Bumjin Lim^3*^, Jung-Hoon Park^2*^

^1^Department of Biosystems & Biomaterials Science and Engineering, Seoul National University, Seoul 08826, Republic of Korea

^2^Biomedical Engineering Research Center, Asan Institute for Life Sciences, Asan Medical Center, 88 Olympic-ro 43-gil, Songpa-gu, Seoul 05505, Republic of Korea

^3^Department of Urology, Asan Medical Center, University of Ulsan College of Medicine, 88 Olympic-ro 43-gil, Songpa-gu, Seoul 05505, Republic of Korea

^†^ These authors contributed equally to this work.

^*^ Correspondence:

Bumjin Lim M.D., Ph.D.^1^ and Jung-Hoon Park, Ph.D.^2^

^1^Department of Urology, Asan Medical Center, University of Ulsan College of Medicine, 88 Olympic-ro 43-gil, Songpa-gu, Seoul 05505, Republic of Korea

Tel: 82-2-3010-1835 Fax: 82-2-476-0090

E-mail: lbj1986@hanmail.net

^2^Biomedical Engineering Research Center, Asan Institute for Life Sciences, Asan Medical Center, 88 Olympic-ro 43-gil, Songpa-gu, Seoul 05505, Republic of Korea

Tel: 82-2-3010-4123 Fax: 82-2-476-0090

E-mail: jhparkz[@amc.seoul.kr](mailto:hyjung@amc.seoul.kr)


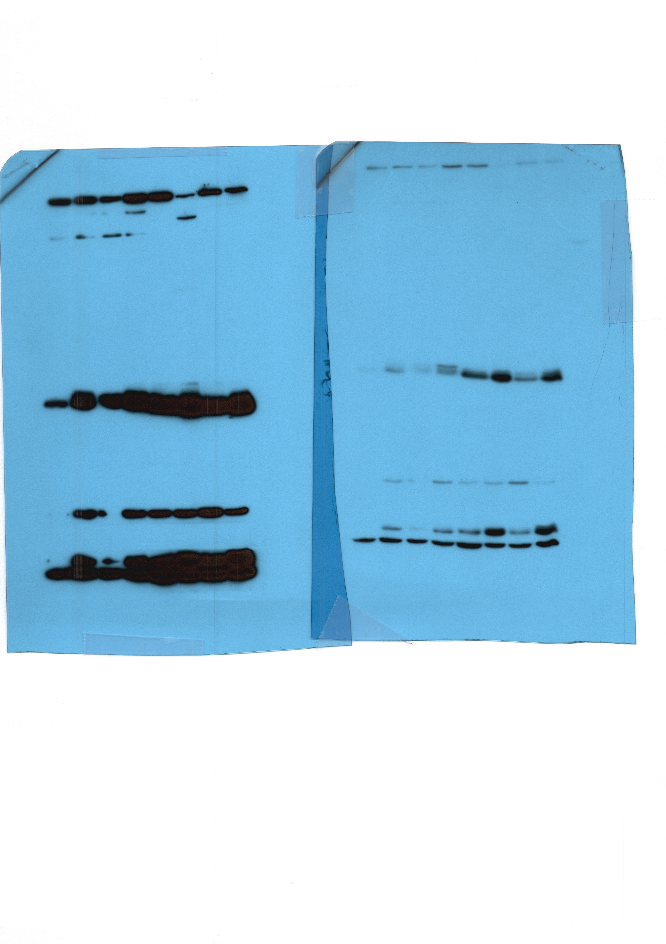


**Supplementary Figure 1.** Full length gels and blots of beta-actin.


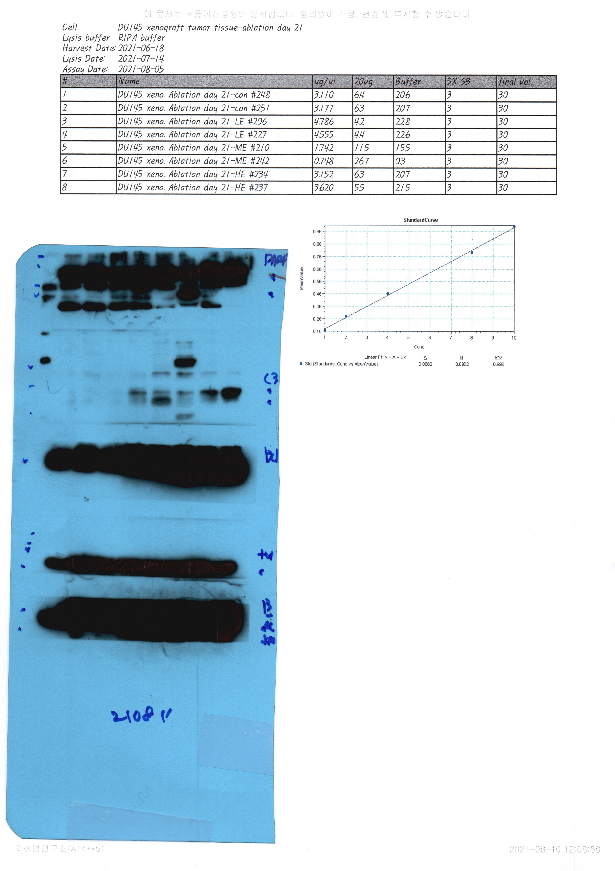


**Supplementary Figure 2.** Full length gels and blots of cleaved caspase-3.
